# Interconversion of Plasma Free Thyroxine Values from Assay Platforms with Different Reference Intervals Using Linear Transformation Methods

**DOI:** 10.3390/biology10010045

**Published:** 2021-01-11

**Authors:** Fanwen Meng, Jacqueline Jonklaas, Melvin Khee-Shing Leow

**Affiliations:** 1 National Healthcare Group, Department of Health Services and Outcomes Research, 3 Fusionopolis Link, Nexus@One-North, Singapore 138543, Singapore; fanwen_meng@nhg.com.sg; 2 Division of Endocrinology, Department of Medicine, Georgetown University, 4000 Reservoir Road NW, Washington, DC 20057, USA; jonklaaj@georgetown.edu; 3 Lee Kong Chian School of Medicine, Nanyang Technological University, 11 Mandalay Road, Singapore 308232, Singapore; 4 Department of Endocrinology, Tan Tock Seng Hospital, 11 Jalan Tan Tock Seng, Singapore 308433, Singapore; 5 Cardiovascular and Metabolic Disorders Program, Duke-NUS Medical School, 8 College Road, Singapore 169857, Singapore; 6 Yong Loo Lin School of Medicine, National University of Singapore, Singapore 119077, Singapore

**Keywords:** reference intervals, assays, scale transformation, equilibrium dialysis, thyroid hormones

## Abstract

**Simple Summary:**

Thyroid hormones are extremely potent and exert a broad range of biological actions on many organ systems of all vertebrates including humans. Blood concentration of thyroid hormones mirrors thyroid status quite well. As thyroid hormone excess or deficiency can lead to serious diseases, it is crucial to ensure that measurement techniques of blood thyroid hormones are accurate and precise, especially during the treatment of an overactive or underactive thyroid. Until recently, many laboratories employ different methods of analysis of thyroid hormones, resulting in reports showing values calibrated to dissimilar normal ranges. This becomes a major issue for patients who are tested in different healthcare facilities as it is challenging to interpret their thyroid status and decide if any difference is due to a real change in hormone concentration or whether the variations occurred purely from calibration differences. In this study, we test the reliability of a mathematical system using linear transformation strategies to convert one value in one scale to another value in a separate scale. Via simultaneously analyzed unbound fraction of plasma thyroxine using three different techniques-immunoassay, mass spectrometry and equilibrium dialysis, we show that linear methods are quite successful in achieving accurate inter-scale thyroid hormone conversions.

**Abstract:**

Clinicians often encounter thyroid function tests (TFT) comprising serum/plasma free thyroxine (FT4) and thyroid stimulating hormone (TSH) measured using different assay platforms during the course of follow-up evaluations which complicates reliable comparison and interpretation of TFT changes. Although interconversion between concentration units is straightforward, the validity of interconversion of FT4/TSH values from one assay platform to another with different reference intervals remains questionable. This study aims to establish an accurate and reliable methodology of interconverting FT4 by any laboratory to an equivalent FT4 value scaled to a reference range of interest via linear transformation methods. As a proof-of-concept, FT4 was simultaneously assayed by direct analog immunoassay, tandem mass spectrometry and equilibrium dialysis. Both linear and piecewise linear transformations proved relatively accurate for FT4 inter-scale conversion. Linear transformation performs better when FT4 are converted from a more accurate to a less accurate assay platform. The converse is true, whereby piecewise linear transformation is superior to linear transformation when converting values from a less accurate method to a more robust assay platform. Such transformations can potentially apply to other biochemical analytes scale conversions, including TSH. This aids interpretation of TFT trends while monitoring the treatment of patients with thyroid disorders.

## 1. Introduction

Clinicians managing thyroid patients often need to monitor thyroid function tests (TFT), comprising serum or plasma free thyroxine (FT4) and thyroid stimulating hormone (TSH), performed serially so as to determine the most appropriate dose adjustments of thyroid medications that will enable the individualized therapeutic targets to be attained in the shortest possible time. Ideally, patients receiving regular follow-up should have their blood tests done at precisely the same time, preferably in the fasted state and analyzed by the same clinical laboratory using identical assay platforms at every single doctor’s visit [1].

In reality, patients may not have their TFT consistently performed in the same medical facility for various reasons [1,2]. It is thus not uncommon to encounter patients with TFT from two or more different laboratories, each reported under a different normal reference range due to the lack of harmonization of test platforms [3]. Under such circumstances, physicians occasionally dismiss such TFT on the grounds that they are incomparable or confounded by inter-assay variations. Patients may then be advised to repeat the TFT in one single laboratory for the purpose of consistency to facilitate interpretation of serial thyroid hormone changes and dose-responses to thyroid medications. However, this approach wastes much past data records, while necessitating additional time, costs, effort, and also leads to the unnecessary pain of repetitive venipunctures. Notwithstanding analytical and biological variability, most TFT assays demonstrate excellent linearity across a broad measurement range [4]. As well, relatively good correlations exist between different TFT assay techniques [5,6]. Hence, we propose two transformation methods (linear and piecewise linear) to interconvert FT4 reported by any laboratory to a specific scale of interest that permits comparisons for time/dosing trends to aid the management of patients with thyroid disorders.

## 2. Materials and Methods

### 2.1. Study Design and Participants

We analyzed 62 paired-samples of TFT (comprising both FT4 and TSH) obtained from overtly healthy individuals aged 18 to 65 years from both genders without any significant past medical history measured using direct analog immunoassay and tandem mass spectrometry compared against equilibrium dialysis simultaneously. None of the females included were pregnant and none of the subjects were sick at the time of the study. All subjects gave their written informed consent for inclusion before they participated in the study. The protocol received ethical approval from the Institutional Review Board of Georgetown University (IRB code 02-412) on 13 March 2003 and this study was conducted in accordance with the Declaration of Helsinki. This TFT dataset were extracted from an anonymized database of research subjects from this study.

### 2.2. Laboratory Measurements

For measurement of FT4 in this study, three methods were simultaneously applied for each subject’s blood sample: direct analog immunoassay (IA), tandem mass spectrometry (LC-MS/MS) and equilibrium dialysis (ED). Instead of the phrase “normal range” to refer to the distribution of levels of any analyte found within the healthy samples in a large population in old terminology, the term “reference interval” is used throughout this article. For ED, the Nichols free T4 kit (Nichols Institute Diagnostics, Catalogue # 30-0652, San Clemente, CA, USA) was used according to the directions provided by the manufacturer [7]. To ensure that ED was robust as a ‘gold standard comparator’, its reference method procedure utilized a dialysis buffer with a biochemical composition closely mimicking the ionic environment of serum/plasma and buffered the samples to a pH of 7.40 prior to dialysis using a device comprising a dialysand/dialysate compartment of identical volume separated by a regenerated cellulose membrane and the system thermostatically controlled to 37.0 °C ± 0.50 °C during dialysis. The tandem mass spectrometry procedure applied was described in our previous work [8,9] and was based on an online extraction/cleaning of the injected samples with subsequent introduction into the mass-spectrometer using a built-in Valco switching valve. 400 μL of the treated plasma ultrafiltrate was injected onto a Supelco LC-18-DB (3.3 cm × 3.0 mm, 3.0 μm particle size) chromatographic column equipped with a Supelco Discovery C-18 (3.0 mm) guard column, where it underwent cleaning with 20% (*v*/*v*) methanol in 5 mmol/L ammonium acetate (pH 4.0) at a flow rate of 0.8 mL/min. After 4 min of cleaning the switching valve was activated, the column was flushed with a water/methanol gradient at a flow rate of 0.6 mL/min, and the samples were introduced into the mass spectrometer [8,9]. We used the Dade Dimension RxL Clinical Chemistry Analyzer (Siemens) for the direct analog FT4 immunoassay that has a reference range of 9–16 pg/mL. The reference interval for FT4 measured with LC-MS/MS is 8–21 pg/mL. The reference interval for FT4 measured with Nichols Institute Kit by equilibrium dialysis was 7–23 pg/mL. The respective intra-assay coefficients of variation (CV) are as follows: IA (CV 0.31), LC-MS/MS (CV 0.39) and ED (CV 0.41). The reported FT4 values were measured in units of pg/mL, which was used in the numerical analysis.

### 2.3. Mathematical Transformation Methods

We first define some notations as follows:

[a, b]: the reference interval of FT4 that is chosen as the scale to be mapped into
[u, v]: the reference interval of FT4 values measured by any specific method (e.g., LC-MS/MS, IA, or ED, etc.)
$x_{\mathrm{original}}$: the FT4 value measured with a specific method with the reference interval [u, v]
$x_{\mathrm{new}}$: the converted FT4 value in the chosen scale with reference interval [a, b].

We study the performance of two mathematical transformations to convert the FT4 data with the reference interval [u, v] into equivalent FT4 data in the reference scale [a, b] of choice [10].

Linear transformation:

(1) $$x_{\mathrm{new}\;}=\;a+\;\frac{b-a}{v-u}\left(x_{\mathrm{original}}-u\right)\;\mathrm{for}\;\mathrm{any}\;\mathrm{raw}\;\mathrm{data}\;x_{\mathrm{original}}.$$

Piecewise linear transformation:

(2) $$x_{\mathrm{new}\;}=\;\{\begin{array}{c} \frac{a}{u}x_{\mathrm{original}},\;\;\;\;\;\;\;\;\;\;\;\;\;\;\;\;\;\;\;\;\;\;\;\;\;\;\;\;\;\;\;\;\;\;\;\;\;\;\;\;\;\;\;\mathrm{for}\;x_{\mathrm{original}}<u,\\ a+\frac{b-a}{v-u}\left(x_{\mathrm{original}}-u\right),\;\;\;\;\;\;\;\;\;\;\;\;\;\;\;\;\;\;\;\;\;\mathrm{for}\;x_{\mathrm{original}}\geq u. \end{array}$$

Linear transformation can be defined geometrically as the equation of a straight line passing through two points (u,a) and (v,b). Piecewise linear transformation is the equation of a two-piece continuous line passing through three points (0, 0), (u,a) and (v,b). Note that linear transformation is monotone increasing while piecewise linear transformation is monotone increasing on each piece [11]. For the subjects with FT4 values in the reference interval of a certain assay method, the underlying transformations ensure that the transformed values will still be in the reference interval in the new scale, and vice versa [11]. FT4 values in the hypothyroid and thyrotoxic ranges of any particular assay should also show correspondingly hypothyroid and thyrotoxic levels when transformed into another assay with a different reference interval. However, the converted value using linear transformation might very occasionally yield negative numbers for some raw FT4 data with respect to certain scales [11]. To overcome this, we impose some condition on the reference interval scale of interest to ensure that all converted values are positive. In practice, the situation of “non-positiveness” is actually uncommon. For our dataset, the FT4 values measured with all the three methods have a relatively strong correlation. Piecewise linear transformation can ensure all interconverted FT4 return positive values regardless of the choice of the scale.

Let [a,b] be the frame of reference and [u,v] be the reference interval of FT4 values measured with a certain method. Then, the transformed FT4 values using linear transformation will be always positive if the following relation holds: $\frac{a}{b}\geq \frac{u}{v}$. For any transformation scale and reference interval under consideration, we may use the inequality condition stated above to identify how well defined is the transformation regarding the positive-ness of converted FT4 values, in addition to detecting outliers of the data.

### 2.4. Statistical Analyses

Mean and standard deviation (SD) were estimated for the three methods. Horn’s method was applied to detect any outliers. We used Shapiro-Wilk test to investigate the normality of the data for each method. 95% reference interval and 90% confidence intervals of lower and upper limits were also computed. Pairwise comparisons of FT4 values obtained with the three assays were performed using the non-parametric Passing-Bablok regression. All the analyses were performed in R3.6.2 (R Core Team (2019). R: A language and environment for statistical computing. R Foundation for Statistical Computing, Vienna, Austria. URL https://www.R-project.org/).

## 3. Results

We analyzed the raw FT4 data measured with LC-MS/MS, IA, and ED of 62 healthy subjects. There were 4 outliers (2 with hyperthyroidism and 2 with hypothyroidism confirmed by the 3 assay methods). The remaining FT4 data of 58 subjects follows a normal distribution (Shapiro-Wilk test *p*-value = 0.249, 0.488, 0.294, respectively).

Table 1 presents estimations of normal intervals for FT4 assays using the full dataset inclusive of outliers. Mean FT4 values obtained with IA were 8.3% and 6.0% lower than values measured with LC-MS/MS and ED, respectively. Mean FT4 values measured with the latter methods differed by approximately 2.5%. Coefficients of variation of the data measured with these methods were 0.39, 0.31 and 0.41. The estimated 95% reference lower limits of these methods are all left-shifted compared with lower boundaries of the population reference ranges provided by the manufacturers as shown in Table 2. However, the estimated 95% reference upper limits of these methods are close to the right boundaries of the population reference ranges. For ED, the reference limits are significantly lower than its population reference interval. For LC-MS/MS and IA, their lower reference limits are significantly lower than the lower boundaries of their population reference intervals. This reflects differences in the FT4 distribution characteristics of our sample from the general population.

**Figure 1:**
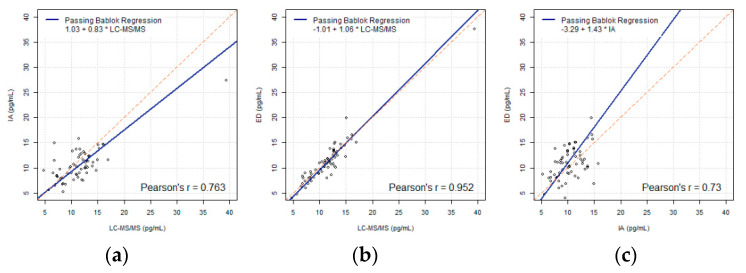
(**a**–**c**) Passing-Bablok regressions of FT4 (LC-MS/MS, IA, ED) using the FT4 data including outliers (*N* = 62), compared pairwise.

There was a strong correlation between FT4 values with IA and either LC-MS/MS (Pearson’s r = 0.763) or ED (Pearson’s r = 0.73) [12]. In particular, FT4 values measured with LC-MS/MS were highly correlated with ED (Pearson’s r = 0.952) with a slope of 1.06 being very close to 1. Using linear regression functions shown in Figure 1A–C, the converted values of the boundary points of each two pairwise reference intervals were inconsistent (i.e., reference interval of one method did not correspond to the reference interval in a different scale after transformation using statistical linear regression functions. Thus, statistical linear regression functions are inappropriate as the transformation function under consideration.

### 3.1. Numerical Analysis on Transformation

Raw data was re-sorted in ascending order for each method. In the reference interval scale for each method, FT4 values with the other two methods were transformed using linear transformation and piecewise linear transformation, respectively. Comparisons on mean, SD, CV of FT4 values before and after the transformation were performed.

#### 3.1.1. Linear Transformation

**Figure 2:**
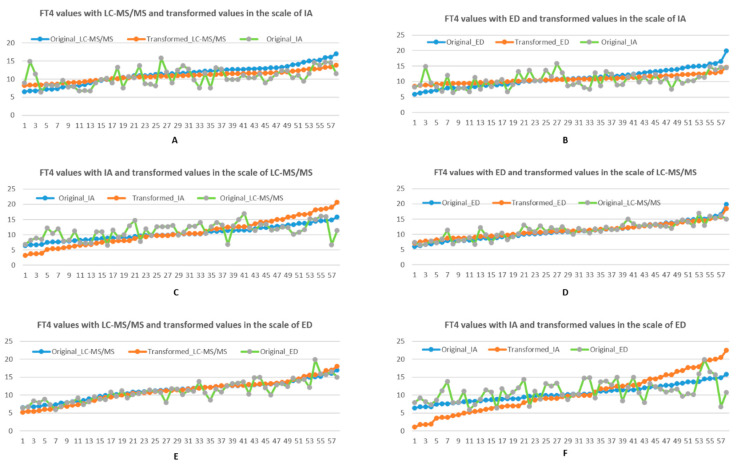
(**A**,**B** top row). Comparison FT4 values and their transformations in the reference interval scale (9, 16) of IA, with respect to LC-MS/MS and ED, using linear transformation. (**C**,**D** middle row). Comparison FT4 values and their transformations in the reference interval scale (8, 21) of LC-MS/MS, with respect to IA and ED respectively, using linear transformation. (**E**,**F** bottom row). Comparison FT4 values and their transformations in the reference interval scale (7, 23) of ED, with respect to LC-MS/MS and IA, using linear transformation. Blue represents original FT4 result, orange represents the transformed scale (from the blue line) to the scale of interest, while green represents the original untransformed results in the scale of interest.

As shown in Table 3, mean, SD and CV are reduced after the transformation into the reference interval scale (9 to 16 pg/mL) of IA. In particular, SD and CV of FT4 values are respectively reduced by over 46% and 43% when transformed from LC-MS/MS to IA. For transformation from ED to IA, the reductions in SD and CV are around 56% and 55%, respectively. Mean values are reduced by 5% and 3% when transforming from LC-MS/MS and ED to IA scales, respectively. Compared to the original curve of FT4 values, Figure 2A,B show that the transformation curves appear to be flat for both LC-MS/MS and ED. The trend of converted values maintained a similar pattern as the original values.

For mapping into the LC-MS/MS scale, Table 4 shows SD and CV of FT4 values converted from IA are increased by over 80%, whereas the SD and CV of FT4 when converted from ED are reduced by approximately 20%. Small changes in mean values between IA and ED upon conversion to the LC-MS/MS scale are about 3% and 2%. Figure 2C,D show that the FT4 curves before and after transformation are almost identical. In converting to the ED scale, Table 5 shows that SD and CV of FT4 values transformed from IA are dramatically increased by 128% and 135%, respectively, as reflected in Figure 2F. In addition, SD and CV when mapped from LC-MS/MS are increased by over 20%. However, there are very small changes on mean values with these two methods. Figure 2E demonstrates the converted FT4 values to ED scale from original LC-MS/MS are very close to the original ones in ED.

#### 3.1.2. Piecewise Linear Transformation

**Figure 3:**
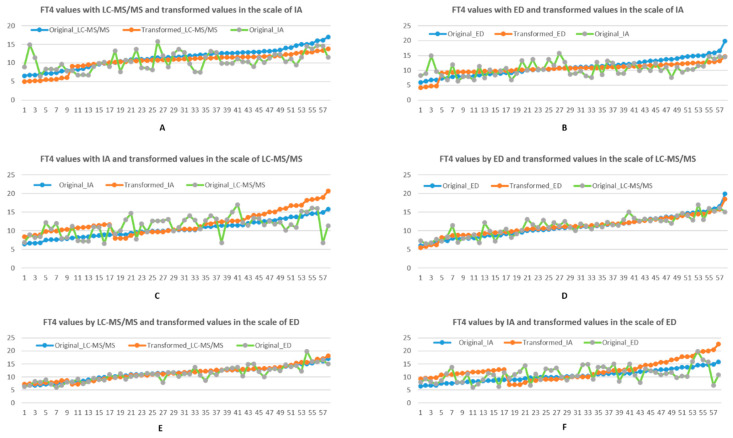
(**A**,**B** top row). Comparison between FT4 values and their transformations in the reference interval scale (9, 16) of IA, with respect to LC-MS/MS and ED, using piecewise linear transformation. (**C**,**D** middle row). FT4 values and their transformations in the reference interval scale (8, 21) of LC-MS/MS, with respect to IA and ED, using piecewise linear transformation. (**E**,**F** bottom row). Comparison between FT4 values and their transformations in the reference interval scale (7, 23) of ED, with respect to LC-MS/MS and ED, using piecewise linear transformation. Blue represents original FT4 result, orange represents the transformed scale (from the blue line) to the scale of interest, while green represents the original untransformed results in the scale of interest.

We conducted similar numerical analysis as above by using piecewise linear transformation. Unlike linear transformation, as shown in Figure 3A–F, the curves of transformed FT4 values are in form of two-piece linear functions. For mapping into the IA reference interval scale, as shown in Table 6, mean, SD, CV of transformed FT4 values from LC-MS/MS are reduced by 9%, 10%, 1.5%. The transformed values from ED are reduced by 5%, 31%, and 27%.

For mapping into the reference interval scale of LC-MS/MS, the mean, SD and CV of transformed values from the IA scale are increased by 15%, 33% and 18%, respectively. However, SD and CV associated with transformation from ED scale are reduced by respectively 11% and 12% while the mean value is slightly increased by 1%. In the reference scale of ED, the transformed values from LC-MS/MS are changed slightly in mean, SD and CV. The conversions from IA are increased by 19%, 55% and 34% respectively.

Generally, assay performance in ascending order of accuracy and precision is IA, followed by LC-MS/MS and ED respectively. Comparing CV and SD changes by linear transformation against piecewise linear transformation as shown in Table 3, Table 4, Table 5, Table 6, Table 7 and Table 8, when a more accurate method (i.e., ED and LC-MS/MS) is transformed to the scale of a less accurate technique (i.e., IA), linear transformation outperformed piecewise transformation. Even between two accurate methods, linear transformation works better than piecewise transformation when highly accurate ED data are transformed into the LC-MS/MS scale. However, when results from a less accurate technique is transformed into the scale of a more accurate technique, piecewise proved far better to linear transformation. For example, when IA results are transformed into the ED scale, the CV change falls from 135% to 34%, if piecewise transformation is applied instead of linear transformation. Similarly, the CV falls from 85% to 18% when mapping IA into the LC-MS/MS scale using piecewise instead of linear transformation.

**Figure 4:**
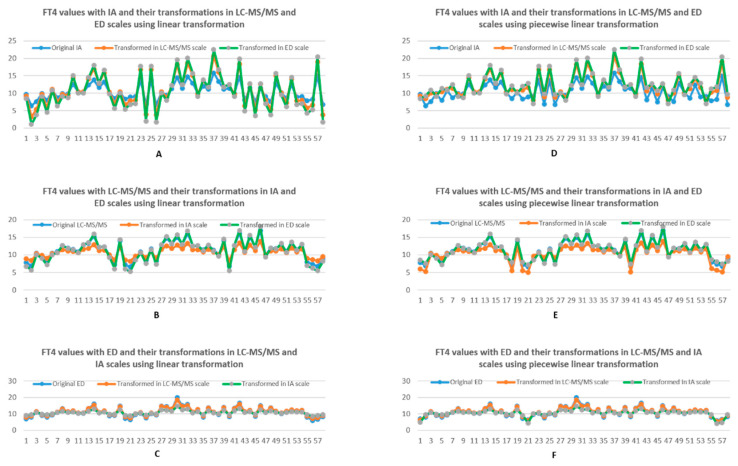
(**A**–**C**) Comparison between FT4 values with one method being the scale of reference and the transformed FT4 values from another two methods, using linear transformation. (**D**–**F**) Comparison between FT4 values with one method being the scale of interest and the transformed FT4 values with another two methods, using piecewise linear transformation. Blue represents the original data, while orange and grey represents corresponding data from two other methods transformed into the scale of the original using piece-wise linear transformation.

As shown in Figure 4A–C, the FT4 values with ED were in the middle of the transformed values with LC-MS/MS and IA in the reference scale of ED. In other words, transformed FT4 values oscillate around the values in ED, particularly with fluctuations associated with IA being larger than LC-MS/MS. There are similar observations in the reference scale of LC-MS/MS as demonstrated in Figure 4B. However, as for FT4 measured in the IA scale, the transformed FT4 values were higher than the original values measured in IA scale for small FT4 values while the transformed values were lower than the scaled value for bigger FT4 values with IA as shown in Figure 4A. These observations facilitate understanding of different FT4 transformation patterns into any specific scale of interest. As to the case of using piecewise linear transformation, as demonstrated in Figure 4D–F, similar patterns can be found in contrast to linear transformation. However, the fluctuations of transformed FT4 values around the scaled FT4 value were considerably reduced in general. There were still non-monotonous trends between the transformed values with the scaled values in IA as shown in Figure 4D.

Table 9 and Table 10 represent illustrative examples of FT4 values measured by IA and corresponding FT4 values transformed into IA from LC-MS/MS and ED scales, using linear transformation and piecewise linear transformation, respectively. These specific examples are especially useful because they allow practicing clinicians to see how the transformation techniques as described above actually work in the real bedside situation, since all the statistical analyses above do not provide much insights as to the clinical utility of such transformation strategies. We have randomly chosen 10 sets of FT4 values from our total patient sample, each FT4 belonging to a unique individual patient measured by 3 assay methods. It can be seen that linear transformation could derive transformed FT4 values from LC-MS/MS and ED which mapped into the IA scale satisfactorily, whereas piecewise linear transformation led to gross under- and over-estimations of the IA scale.

## 4. Discussion

FT4 is a very commonly ordered test but one unfortunately plagued by many factors that impact on its measurement accuracy and precision. Excluding non-analyte related interferences such as heterophil antibodies and those inherently linked to biotin-streptavidin system weaknesses, current immunoassay methods suffer from weaknesses due to both intra-assay variations and “between-method” biases [13,14,15,16,17,18,19]. Until now, the persistent reliance on different methodologies (e.g., IA, LC-MS/MS, ED) for FT4 measurement together with significant “between-method” platform variability and biases far exceeding biological variations meant that no universal normal population reference ranges applicable across all methods currently exist. This is the very reason motivating the International Federation of Clinical Chemistry (IFCC) committee to move towards the standardization of TFT (C-STFT) [20,21,22,23,24,25]. While some progress in terms of re-standardization of TFTs against established reference measurement procedures have occurred, the harmonization and recalibration have yet to be adopted and implemented by those within the in-vitro diagnostic industries after all these decades [26].

To complicate matters, there are still patients with thyroid disorders who continue to have their TFTs done at different medical facilities and who routinely show their physicians laboratory reports that have FT4/TSH readings in different units and/or with different normal population ranges. Occasionally, laboratories of hospitals have been known to switch assay methodologies for various valid reasons that affect the continuity of follow-up comparisons of TFTs. As such, medical doctors attending to patients with thyroid conditions are often confronted with challenges that include the interpretation of a mixture of FT4/TSH results emerging from different assays. Since it is challenging to dissect differences in FT4 values contributed by underlying disease processes from variations attributed to assay platforms biases, it is very helpful to develop robust and reliable techniques of interconverting these FT4 values from different laboratory reports to a common scale of choice and reference range to render interpretation of trends and diagnosis possible. In this present study, we have only confined ourselves to the development of an interconversion method for FT4 for the sake of illustrating a proof-of-concept. Such a method should plausibly apply to the interconversion of TSH or other biochemical analytes as well. We wish to emphasize here that the goal of this paper is not about the validation, standardization and harmonization of different assay methods of FT4 and TSH. That is the effort and work of the committee of the IFCC C-STFT [20,21,22,23,24,25]. Our present dissertation is categorically not about trying to replicate what has elegantly been established as the current standardization status of FT4/TSH immunoassays. Instead, we are proposing a method to assist clinicians to cope with individual patients’ results of FT4 reported by different laboratories showing different reference intervals so that it is possible to make objective comparisons and allow meaningful interpretation of the trend in FT4 over time.

With the availability of FT4 data emerging from accurate assay methods at our disposal (i.e., LC-MS/MS and ED), we are able to prove via numerical analysis that both techniques of interconversions performed reasonably well, with linear transformation showing better accuracy when FT4 values from a more accurate assay method are converted to a scale of a less accurate method. This helps doctors to make meaningful comparisons between results from laboratories with different assay platforms and clinical reference ranges. Moreover, the proposed transformation techniques can potentially apply to other analytes scale conversions, such as TSH, though this was not formally shown in our study due to the lack of simultaneously measured TSH using different assay platforms. Notwithstanding the logarithmic-linear relationship of TSH-FT4 [27,28,29,30,31], because many TSH assays generally exhibit a linear assay dynamic range, this implies that it is likely amenable to such linear transformation methods for interconversion [15,32].

As an example, we consider a hypothetical typical clinical scenario of a patient referred by his/her primary care physician to an endocrinologist to assist with fine-tuning of his/her L-thyroxine (LT4) dose. This patient produced a set of TFT done at his/her primary physician’s laboratory as follows. While being prescribed a dose of LT4 of 75 mcg daily, his/her FT4 = 12.8 pg/mL (reference interval lab #1: 9.9–15.8) and his/her TSH = 7.73 mIU/L (reference interval lab #1: 0.70–4.28). This led to some cold intolerance and low energy levels, so he/she decided to double the dose himself/herself to LT4 of 150 mcg daily. By the time he/she consulted the endocrinologist 8 weeks later, a repeat TFT done in this second laboratory yielded these results: FT4 = 18.3 pg/mL (reference interval lab #2: 7.0–19.4); TSH = 0.56 mIU/L (reference interval lab #2: 0.4–4.7). In order to make a fair comparison and more accurately define the magnitude of the changes, the following linear transformation of the patient’s TFT measured in the first laboratory (while on LT4 of 75 mcg od) was performed by the endocrinologist as such:

FT4_new_ = 7 + {[(19.4 − 7.0)/(15.8 − 9.9)] × (12.8 − 9.9)} = 13.1 pg/mL (ref range of lab#2: 7.0–19.4)

TSH_new_ = 0.4 + {[(4.7 − 0.4)/(4.28 − 0.70)] × (7.73 − 0.70)} = 8.84 mIU/L (ref range of lab#2: 0.4–4.7)

Based on the appropriately scaled TFT, the patient’s FT4 had risen considerably from 13.1 pg/mL to 18.3 pg/mL while his/her TSH had declined precipitously from 8.84 mIU/L to 0.56 mIU/L. This endocrinologist could then confidently reduce his/her LT4 dose from 150 mcg to 100 mcg daily. Such is the clinical utility of mathematical scaled transformations at the bedside.

We believe that our methods proposed can help many clinicians managing patients with thyroid diseases by allowing them to convert any FT4 value on any laboratory report to a scale of their choice so as to facilitate comparison and trending of FT4 results. The mathematical formulae to do this are relatively easy to apply at the bedside. However, to simplify the process even further, the linear mathematical transformations can be built into a mobile app that any doctor can rapidly use in the clinics or hospitals to make this interconversion an extremely effortless process.

### Limitations

For grossly deranged FT4 values that far exceed the ranges encountered in our dataset, some assays can increase in an exponential fashion that would not fit a linear strategy of scale transformation. In such extreme instances, regardless of the types of assays used or the scales the results are reported in, the therapeutic decisions required are clinically obvious (e.g., very high or low FT4 values) and not impacted by inaccuracies with the transformation method at those ranges. Hence, the necessity for scale transformation is not critical for grossly abnormal FT4 values because the clinical decisions are relatively standardized. Thus, any lack of non-linearity for FT4 in these pathological ranges does not impact on clinical management. However, stable thyroid patients whose optimal wellbeing can prove rather sensitive to FT4 changes within or slightly outside the normal population ranges continue to pose challenges during serial clinical follow-up. Our linear transformation methods proposed here should be sufficiently accurate and reliable to handle such orders of magnitude of FT4 alterations.

## 5. Conclusions

Linear transformation seamlessly interconverts FT4 values between different assays well when using an appropriate reference interval as the scale. Piecewise linear transformation might be an alternative if linear transformation should result in negative values.

## Figures and Tables

**Table 1:** Basic statistics of FT4 data (pg/mL) inclusive of outliers (*N* = 62) using 3 methods.

Method	LC-MS/MS		IA		ED	
Mean (SD)	11.55 (4.55)		10.58 (3.28)		11.26 (4.57)	
CV	0.39		0.31		0.41	
95% Reference limit	(2.64, 20.46)		(4.16, 17)		(2.31, 20.22)	
90% Confidence interval of limits	(1.02, 4.26)	(18.84, 22.09)	(2.99, 5.33)	(15.84, 18.17)	(0.68, 3.94)	(18.59, 21.85)

**Table 2:** Population reference intervals (pg/mL) for FT4 with three methods.

Method	LC-MS/MS	IA	ED
Reference interval	(8, 21)	(9, 16)	(7, 23)

**Table 3:** Mean, SD and CV of FT4 transformations into IA scale by linear transformation.

Method	LC-MS/MS			ED		
	Original	New	Change%	Original	New	Change%
Mean	11.34	10.80	−4.76%	11.09	10.79	−2.71%
SD	2.61	1.41	−46.08%	2.88	1.26	−56.19%
CV	0.23	0.13	−43.34%	0.26	0.12	−55.03%

**Table 4:** Mean, SD and CV of FT4 transformations into LC-MS/MS scale by linear transformation.

Method	IA			ED		
	Original	New	Change%	Original	New	Change%
Mean	10.49	10.77	2.67%	11.09	11.32	2.11%
SD	2.36	4.38	85.40%	2.88	2.34	−18.65%
CV	0.22	0.41	84.66%	0.26	0.21	−20.42%

**Table 5:** Mean, SD and CV of FT4 transformations into ED scale by linear transformation.

Method	LC-MS/MS			IA		
	Original	New	Change%	Original	New	Change%
Mean	11.34	11.11	−1.99%	10.49	10.41	−0.77%
SD	2.61	3.22	23.26%	2.36	5.39	128.19%
CV	0.23	0.29	25.85%	0.22	0.52	135.15%

**Table 6:** Mean, SD and CV of FT4 transformations into the IA scale by piecewise linear transformation.

Method	LC-MS/MS			ED		
	Original	New	Change%	Original	New	Change%
Mean	11.34	10.32	−8.99%	11.09	10.50	−5.36%
SD	2.61	2.34	−10.41%	2.88	2.00	−30.62%
CV	0.23	0.227	−1.49%	0.26	0.19	−26.78%

**Table 7:** Mean, SD and CV of FT4 transformations into the LC-MS/MS scale by piecewise linear transformation.

Method	IA			ED		
	Original	New	Change%	Original	New	Change%
Mean	10.49	12.07	15.10%	11.09	11.21	1.07%
SD	2.36	3.15	33.30%	2.88	2.56	−11.16%
CV	0.22	0.26	18.43%	0.26	0.23	−12.21%

**Table 8:** Mean, SD and CV of FT4 transformations into the ED scale by piecewise linear transformation.

Method	LC-MS/MS			IA		
	Original	New	Change%	Original	New	Change%
Mean	11.34	11.41	0.58%	10.49	12.44	18.59%
SD	2.61	2.79	6.71%	2.36	3.66	55.24%
CV	0.23	0.24	6.17%	0.22	0.29	33.87%

**Table 9:** An example of FT4 values (pg/mL) measured by IA and transformations in LC-MS/MS and ED scales using linear transformation.

Subject S/N	Original IA	LC-MS/MS to IA	ED to IA	Original LC-MS/MS	Original ED
2	6.40	8.40	9.42	6.89	7.97
58	7.80	8.97	9.42	7.94	7.97
59	8.24	8.64	8.55	7.33	5.96
22	8.30	8.57	9.12	7.21	7.29
20	8.40	8.56	9.82	7.19	8.88
23	8.90	8.22	8.71	6.55	6.35
1	9.65	8.90	8.92	7.81	6.82
52	10.10	11.75	10.34	13.10	10.07
42	11.40	8.34	9.59	6.77	8.35
60	14.90	8.32	8.90	6.75	6.77

**Table 10:** An example of FT4 values (pg/mL) measured by IA and their transformations in LC-MS/MS and ED scales using piecewise linear transformation.

Subject S/N	Original IA	LC-MS/MS to IA	ED to IA	Original LC-MS/MS	Original ED
2	6.40	5.25	9.42	6.89	7.97
58	7.80	6.05	9.42	7.94	7.97
59	8.24	5.58	4.15	7.33	5.96
22	8.30	5.49	9.12	7.21	7.29
20	8.40	5.48	9.82	7.19	8.88
23	8.90	4.99	4.42	6.55	6.35
1	9.65	5.95	4.74	7.81	6.82
52	10.10	11.75	10.34	13.10	10.07
42	11.40	5.16	9.59	6.77	8.35
60	14.90	5.14	4.71	6.75	6.77

## Data Availability

The data presented in this study are available on reasonable request from the corresponding author. The data are not publicly available as they are the asset of the institution and approval for release is controlled by the institution’s data custodian.
